# Clustering of Cardiovascular Risk Factors in Midlife Forms Trajectories That Affect Heart Aging Biomarker and Incidence of Cardiovascular Disease

**DOI:** 10.1016/j.mcpdig.2026.100425

**Published:** 2026-10-05

**Authors:** Arya Panthalanickal Vijayakumar, Tom Wilsgaard, Henrik Schirmer, Haakon Lindekleiv, Zachi I. Attia, Francisco Lopez-Jimenez, David A. Leon, Olena Iakunchykova

**Affiliations:** a Department of Community Medicine, UiT The Arctic University of Norway, Tromsø, Norway; b Akershus University Hospital, Lørenskog, Norway; c Institute of Clinical Medicine, Campus Ahus, University of Oslo, Norway; d Department of Psychology, University of Oslo, Norway; e University Hospital of North Norway, Tromsø, Norway; f Department of Cardiovascular Medicine and Mayo Clinic College of Medicine, Mayo Clinic, Rochester, MN; g Department of Noncommunicable Disease Epidemiology, London School of Hygiene & Tropical Medicine, London, United Kingdom

## Abstract

**Objective:**

To (1) identify multivariable longitudinal midlife patterns of systolic and diastolic blood pressure, body mass index, and non–high density lipoprotein cholesterol and (2) assess whether electrocardiogram derived heart age acceleration (δ-age) mediates their associations with incident myocardial infarction (MI), stroke, and all-cause mortality.

**Patients and Methods:**

We analyzed 5244 participants from the observational cohort study, the Tromsø Study, who were free of MI or stroke at baseline (Tromsø7, 2015-2016) and attended at least 3 surveys (Tromsø3-7, 1986-2016). Age-standardized risk factor *z*-scores were modeled with quadratic B-splines and grouped using K-means clustering to identify joint multivariable life-course risk profiles. δ-Age was derived from 12-lead electrocardiograms using a validated neural network. Natural-effects models decomposed hazard ratios between clusters and clinical events (follow-up to 2021) into direct and δ-age mediated components.

**Results:**

Four distinct clusters were identified: stable low-risk (cluster 1, n=1604, 30.6%), dyslipidemia (cluster 2, n=1340, 25.6%), hypertension (cluster 3, n=1476, 28.1%), and high-risk trajectory (cluster 4, n=824, 15.7%). Each 1-SD increment in δ-age was associated with 27% to 30% of excess MI, stroke, and death, independent of age and sex. Clusters 3 and 4 carried the greatest excess risk of clinical events, whereas mediation by δ-age was modest (4%-8% of total effect for MI and stroke) and not statistically significant.

**Conclusion:**

Joint multivariable life-course clustering of conventional risk factors identified clinically distinct long-term cardiovascular risk phenotypes. Although δ-age adds independent prognostic information, it explained little of the excess risk associated with chronic hypertension, dyslipidemia, or obesity, indicating it functions primarily as a parallel marker and not a causal mediator.

## Graphical abstract

**Figure:**
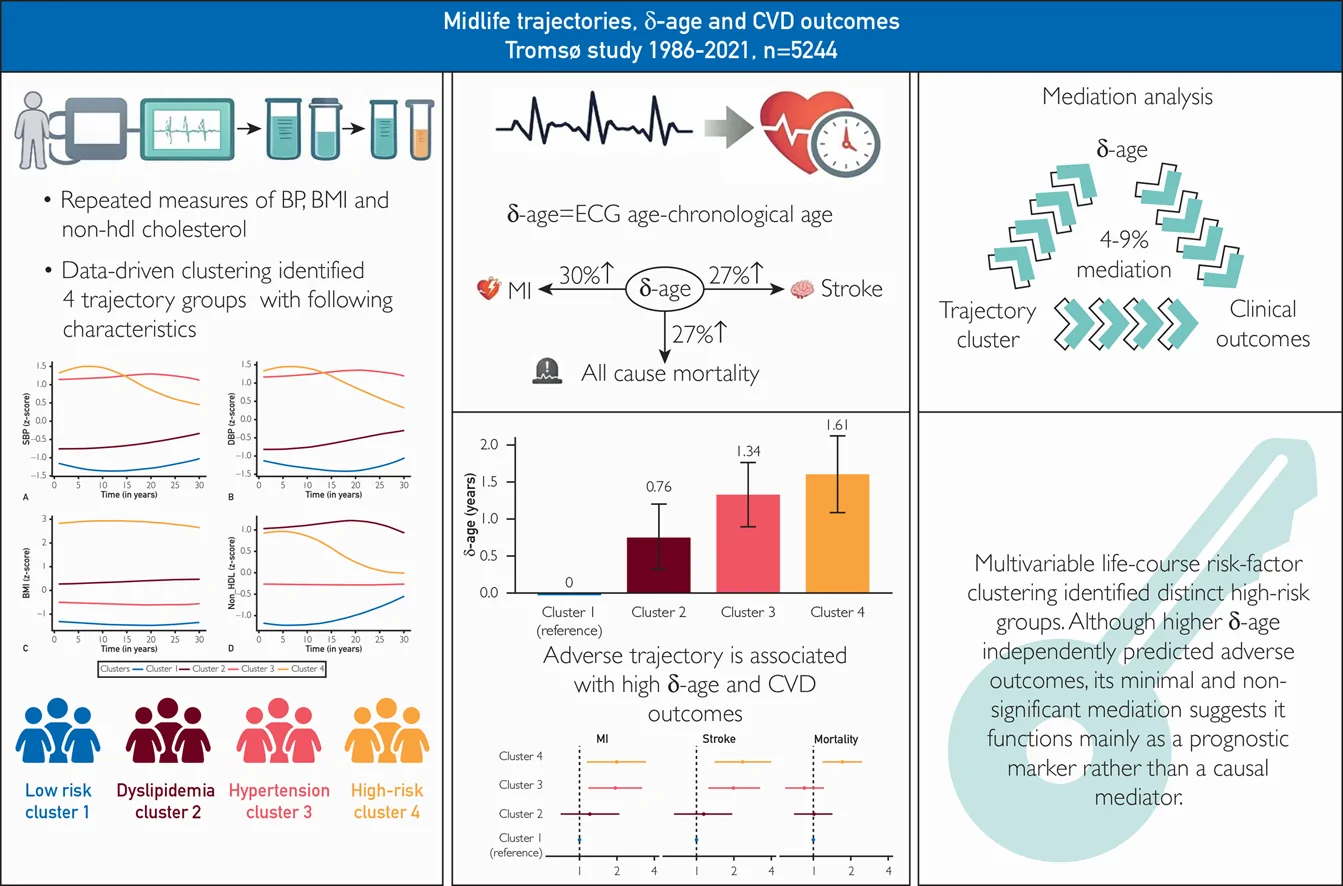

Age stands unrivaled as the main risk factor of chronic illness in humans, and nowhere is this more apparent than in cardiovascular disease (CVD), which remains the leading cause of premature death worldwide, accounting for approximately 18 million deaths every year.^1^ Chronological age captures the passage of time, and it only crudely reflects the biological wear that accumulates within tissues and organs as people age, and the cardiovascular health of people born in the same year can diverge markedly. A diverse toolkit of biological aging metrics has emerged,^2^, ^3^, ^4^ which can inform personalized prognosis, facilitate early detection of age-related disease, and guide tailored interventions.^5^, ^6^, ^7^

Among organ-specific measures, electrocardiogram (ECG)-based heart age provides a clinically intuitive read-out of cardiovascular aging. It is derived from the standard 12-lead ECG processed in a deep convolutional neural network (CNN) that has been trained to predict the heart’s biological age.^8^ Accelerated heart age (δ-age) is the discrepancy between algorithm-derived ECG-based age and chronological age. We and others^9^, ^10^, ^11^ hypothesize that this biomarker translates decades of subclinical damage into a single, readily interpretable number. Large-scale cohorts show that each 1-year increase in δ-age independently raises the risk of CVD events and all-cause mortality, even after comprehensive adjustment for conventional risk factors.^10^, ^12^, ^13^, ^14^, ^15^ Nonetheless, it is still uncertain whether δ-age is simply a marker of accumulated damage or instead serves as an important mediating pathway linking long-term cardiometabolic exposures to clinical events.

Unraveling this requires longitudinal data because repeated measurements could reveal heterogeneous trajectories that single time points obscure. Unsupervised, data-driven clustering condenses such trajectories into discrete risk profiles; however, only a few studies have investigated whether such profiles carry prognostic information above and beyond baseline risk levels.^16^, ^17^ Although joint life-course clustering of conventional risk factors has been linked to cardiometabolic disease and ECG-derived heart age predicts hard cardiovascular end points, no study has, to our knowledge, integrated these approaches. A formal causal-mediation test of whether ECG-derived δ-age itself lies on the pathway from long-term, jointly clustered risk factor exposure to hard end points has not been performed. It, therefore, remains unresolved whether δ-age is a potential treatment target or a parallel prognostic marker.

Using 3 decades of follow-up from the community-based Tromsø Study, we (i) delineate distinct longitudinal cardiovascular risk profiles through unsupervised clustering of repeated measurements of several conventional risk factors jointly and (ii) test whether δ-age mediates the associations between these multivariable trajectory-defined profiles and subsequent myocardial infarction (MI), stroke, and all-cause mortality (Graphical Abstract).

## Patients and Methods

### Study Design and Sample

The Tromsø Study is a population-based study conducted since 1974 in the municipality of Tromsø, Norway. It consists of 7 waves, known as Tromsø1 through Tromsø7, with a total of 45,473 people attending 1 or more surveys. Questionnaires, biological samples, and health examinations were used to collect data. The present analysis includes data collected from 5 waves of the Tromsø Study (1986-2016), including Tromsø3 (1986-1987), Tromsø4 (1994-1995), Tromsø5 (2001), Tromsø6 (2007-2008), and Tromsø7 (2015-2016).^18^, ^19^ Tromsø4 to 7 also included a second, more extensive examination of the participants. In Tromsø7, 8346 participants attended this follow-up visit, and 7778 of them underwent a 12-lead ECG examination. Of these, 5770 participants have attended at least 3 of 5 waves and 28 excluded because of 1 or more missing values in the measurements of systolic and diastolic blood pressure (BP) values, body mass index (BMI), and total and high-density lipoprotein (HDL) cholesterol. Excluding participants with prevalent MI (n=349), stroke (n=179), including 30 with bothbefore Tromsø7 (baseline) left a final sample of 5244.

The Tromsø Study complies with the Declaration of Helsinki and has been approved by the Regional Committee for Medical and Health Research Ethics (REK), the Data Inspectorate, and the Norwegian Directorate of Health. All participants provided written informed consent. The current study was approved by the REK Nord (reference 68185), and a data management plan was evaluated by the Norwegian Centre for Research Data.

### Measurements

#### CVD Risk Factors and δ-Age

Body mass index was computed as weight divided by height squared. Blood pressure was measured 3 times with an oscillometric device after a 2-minute seated rest; the mean of the 2 final readings was used. Serum total and HDL cholesterol were analyzed by enzymatic colorimetric methods in nonfasting venous blood samples. The devices and assay platforms for each survey are given in the Supplemental Appendix 1 (available online at http://www.mcpdigitalhealth.org/). Non-HDL cholesterol was calculated as the difference between total and HDL cholesterol.

In the second visit of Tromsø7, 12-lead resting ECGs were recorded with a computer-based electrocardiograph (Cardiovit AT-104 PC; Schiller AG). To estimate ECG-based age, these digital ECGs were directly input into a pretrained CNN algorithm developed and trained by our team using Mayo Clinic data.^8^ The δ-age was calculated as the difference between the CNN-derived ECG age and chronological age.

#### Clinical end Points

Prevalent cases at baseline (Tromsø7, 2015-2016) and incident cases of MI and stroke until December 31, 2021, were recorded from multiple sources. Events up to 2014 came from the Tromsø Study’s local CVD register,^20^, ^21^ whereas those from 2015 to 2021 were obtained from national registries of MI and stroke. Registry linkage procedures and diagnostic inclusion criteria are given in the Supplemental Appendix 1. The National Cause of Death Registry from the Norwegian Institute of Public Health supplied all-cause mortality data through the end of 2021. National registers records overlapped with the local register for 2013 to 2014 and have been verified complete and in agreement with the validated diagnoses in the local register.^22^, ^23^

### Statistical Analyses

The statistical analyses were performed in 2 sequential stages: (1) derivation of longitudinal cardiovascular risk factor trajectories and (2) assessment of their associations with cardiovascular outcomes and the mediating role of δ-age.

#### Standardization of Risk Factor Variables

For each examination round (Tromsø 3-7), systolic BP, diastolic BP, BMI and non-HDL cholesterol were converted to age-specific standardized score (*z*-scores). Age standardization used fractional polynomial regression^24^ of chronological age, with the model for each variable and wave chosen by the minimum Akaike information criterion (Supplemental Appendix 1). Descriptive characteristics are presented as mean (SD) for continuous CVD risk factors and number (percentage) for sex.

#### Derivation of Multivariable Trajectories

Within each participant, the 4 standardized variables were modeled simultaneously with quadratic B-splines. Ordinary least squares estimated the spline coefficients that best reproduced the observed measurements. Coefficients from all longitudinal variables were concatenated to form a single trajectory vector per person. These vectors were clustered into 4 initial trajectory clusters and optimal clusters were estimated with a K-means algorithm.^25^ The update, reallocation, and convergence rules of the cluster are given in the Supplemental Methods.

#### Association of Trajectory Cluster With Clinical End Points and Mediation by δ-Age

We used linear regression to examine the association between trajectory clusters and δ-age. For time-to-event analyses, we fitted Cox proportional hazard regression models, with time scale defined as the duration (years) from date of attendance at Tromsø7 to the event date or to censoring at death from other causes, emigration, or end of follow-up (December 31, 2021), to assess the association between trajectory clusters or δ-age with 3 outcomes: incident MI, incident stroke, and all-cause mortality, adjusted for sex and chronological age. For each end point, natural-effects mediation models for survival data^26^ were fitted to quantify the indirect effect (IE) operating through δ-age. These models provided hazard ratios (HRs) for the natural direct effect (DE), IE, and total effect (TE). The proportion mediated was calculated on the log-HR scale as ln(IE)/ln(TE).^26^ The 95% CIs were obtained from 1000 nonparametric bootstrap resamples. The proportional hazards assumption was verified for each Cox model using scaled Schoenfeld residuals.

Mediation by δ-age in the association between risk factor trajectories and events was further investigated in supplementary analyses, one by using age-standardized quadratic B-spline curves derived clusters separately for systolic BP, diastolic BP, body mass index, and non-HDL cholesterol over 30 years of exposure. The second analysis examined mediation by δ-age for associations between a single risk factor measured at a single time point (Tromsø7) and incident clinical outcomes, and this was repeated for all risk factors. Then, finally, by changing the number of clusters to 3 and 5, followed by the mediation analysis. These analyses were used to better understand the role of δ-age and to assess the robustness of the findings relative to the main analysis.

## Results

### Identification of Trajectory Clusters

The K-means algorithm identified four distinct longitudinal patterns (Figure 1) in the age-standardized trajectories of risk factors, including systolic BP, diastolic BP, BMI, and non-HDL cholesterol. The mean and SD for each risk factor by cluster and time points are shown in Table 1. Cluster 1 was characterized by substantially low values for all risk factors, thereby being the stable low-risk cluster, comprised 1604 (30.6%) participants and was characterized by persistently favorable levels across all 4 risk factors on all time points. Cluster 2 (n=1340, 25.6%) was characterized by consistently raised non-HDL cholesterol with only modest deviations in BP and BMI. Cluster 3 (n=1476, 28.1%) was characterized by elevated systolic and diastolic BPs over the entire follow-up and can be marked as the hypertensive cluster. Finally, cluster 4 (n=824, 15.7%) was characterized by chronically elevated BMI and BP, both systolic and diastolic (similar to cluster 3 in BP pattern), with the second highest mean for lipids marking as a high-risk cluster. Across all clusters, the mean participant age was approximately 66 to 67 years at the time of ECG examination (the second visit of Tromsø7). However, the proportion of women was higher in the stable low-risk cluster (cluster 1, n=1233, 76.9%) and lower in the high-risk cluster (cluster 4, n=361, 43.8%).

**Figure 1:**
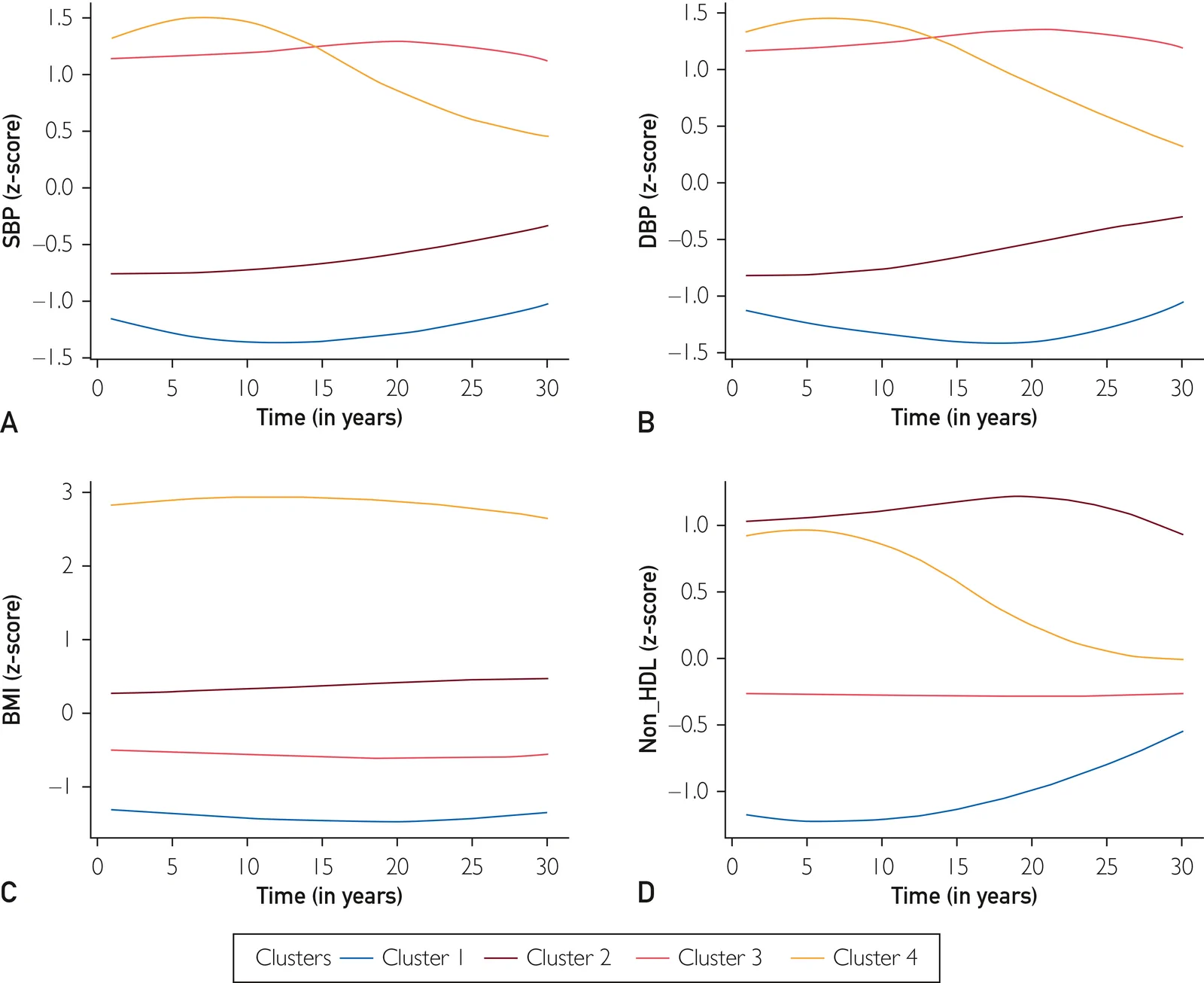
Age-standardized (z-score) B-spline trajectories of cardiovascular risk factors for the 4 identified joint multivariable trajectory clusters, The Tromsø Study 1986-2016: (A) systolic blood pressure (SBP); (B) diastolic blood pressure (DBP); (C) body mass index (BMI); and (D) non–high-density lipoprotein (HDL) cholesterol.

**Table 1:** Descriptive Statistics by 4 Multivariable 30-Year Risk Factor Trajectory Clusters (2015-2016): The Tromsø Study 1986-2016

Variable^a^	Study^b^	Cluster 1 (low-risk cluster) (1604, 30.6%)	Cluster 2 (dyslipidemia cluster) (1340, 25.6%)	Cluster 3 (hypertensive cluster) (1476, 28.1%)	Cluster 4 (high-risk cluster) (824, 15.7%)
Age (y)	Tromsø7	66.1 (8.1)	67.3 (8.2)	67.1 (8.2)	66.7 (8.2)
Women, n (%)	Tromsø7	1233 (76.9)	766 (57.2)	691 (46.8)	361 (43.8)
BMI (kg/m^2^)	Tromsø3	21.68 (1.96)	24.14 (2.20)	22.99 (2.07)	28.00 (2.98)
	Tromsø4	22.55 (2.13)	25.68 (2.34)	24.16 (2.17)	30.11 (3.05)
	Tromsø5	23.58 (2.48)	27.32 (2.68)	25.31 (2.40)	32.46 (3.52)
	Tromsø6	23.91 (2.60)	27.85 (2.85)	25.74 (2.45)	32.77 (3.53)
	Tromsø7	24.38 (2.95)	28.29 (3.19)	26.07 (2.82)	33.07 (4.00)
DBP (mm Hg)	Tromsø3	69.61 (7.28)	71.46 (7.31)	81.01 (8.73)	81.38 (9.73)
	Tromsø4	69.88 (7.53)	73.20 (7.84)	83.96 (9.17)	84.78 (10.32)
	Tromsø5	71.31 (7.81)	75.76 (7.63)	86.34 (9.57)	85.39 (11.09)
	Tromsø6	70.79 (7.96)	75.56 (7.79)	85.59 (9.30)	82.29 (9.31)
	Tromsø7	70.27 (8.26)	74.20 (8.26)	81.82 (8.82)	77.59 (9.77)
SBP (mm Hg)	Tromsø3	116.66 (9.69)	119.55 (9.55)	132.99 (12.34)	133.65 (12.52)
	Tromsø4	119.39 (10.28)	124.74 (10.26)	139.62 (14.99)	141.50 (14.87)
	Tromsø5	120.80 (14.44)	128.04 (13.57)	146.13 (17.84)	144.06 (17.92)
	Tromsø6	123.14 (17.09)	132.01 (17.09)	150.52 (19.63)	144.84 (19.11)
	Tromsø7	125.04 (17.60)	133.31 (18.03)	147.25 (18.47)	140.76 (19.17)
Non-HDL (mmol/L)	Tromsø3	3.40 (0.89)	4.70 (1.10)	3.98 (1.09)	4.61 (1.18)
	Tromsø4	3.65 (0.94)	5.15 (1.09)	4.31 (1.09)	4.97 (1.17)
	Tromsø5	4.01 (0.91)	5.43 (1.03)	4.52 (1.01)	5.01 (1.05)
	Tromsø6	3.67 (0.86)	4.90 (0.96)	4.08 (0.94)	4.33 (0.98)
	Tromsø7	3.61 (0.91)	4.50 (1.09)	3.79 (0.97)	3.95 (1.08)

All participants were classified into 1 of 4 trajectory clusters derived by K-means on second-degree B-spline coefficients summarizing 30-year joint patterns of BMI, SBP, DBP and non-HDL.

BMI, body mass index; DBP, diastolic blood pressure; SBP, systolic blood pressure; HDL, high-density lipoprotein.

a Values are mean (SD) or number (%).

b Tromsø3 (1986-1987), Tromsø4 (1994-1995), Tromsø5 (2001), Tromsø6 (2007-2008), and Tromsø7 (2015-2016).

Crude event rates during follow-up are shown in Table 2, with cumulative incidence in Supplemental Figure 1 (available online at http://www.mcpiqojournal.org); MI occurred in 1.4% (n=22) of the low-risk stable cluster 1, in 2.2% (n=29) of the dyslipidemia cluster 2, in 3.8% (n=56) in the hypertensive cluster 3, and in 3.6% (n=30) of the high-risk trajectory cluster 4. Moreover, stroke was registered in 1.6% (n=25), 2.0% (n=27), 3.5% (n=51), and 3.8% (n=31) of participants, and all-cause mortality amounted to 3.6% (n=58), 4.4% (n=59), 3.9% (n=57), and 7.0% (n=58), respectively.

**Table 2:** Natural-Effect Decomposition^a^ of the Association Between Multivariable Trajectory Clusters and Incident Cardiovascular Outcomes, With δ-Age^b^ Specified as the Mediator: The Tromsø Study 1986-2016, Follow-up of Clinical Outcomes Until 2021

Outcome	Cluster	Events, n (%)	IE^c^ (95% CI)	DE^c^ (95% CI)	TE^c^ (95% CI)	Proportion mediated (%)
Myocardial infarction	1	22 (1.4)	1 (Reference)	1 (Reference)	1 (Reference)	Reference
	2	29 (2.2)	1.01 (0.94-1.09)	1.21 (0.69-2.24)	1.22 (0.69-2.22)	5
	3	56 (3.8)	1.04 (0.97-1.12)	1.89 (1.13-3.42)	1.96 (1.19-3.56)	6
	4	30 (3.6)	1.05 (0.97-1.15)	1.89 (1.08-3.60)	1.99 (1.13-3.78)	7
Stroke	1	25 (1.6)	1 (Reference)	1 (Reference)	1 (Reference)	Reference
	2	27 (2.0)	1.01 (0.94-1.08)	1.13 (0.62-1.99)	1.14 (0.63-2.00)	8
	3	51 (3.5)	1.04 (0.96-1.12)	1.97 (1.19-3.40)	2.04 (1.22-3.53)	6
	4	31 (3.8)	1.04 (0.95-1.13)	2.32 (1.42-4.17)	2.40 (1.44-4.31)	4
All-cause mortality	1	58 (3.6)	1 (Reference)	1 (Reference)	1 (Reference)	Reference
	2	59 (4.4)	1.01 (0.96-1.06)	0.99 (0.69-1.44)	1.00 (0.70-1.45)	NA^d^
	3	57 (3.9)	1.04 (0.99-1.10)	0.82 (0.58-1.15)	0.85 (0.60-1.20)	NA
	4	58 (7.0)	1.05 (0.99-1.11)	1.65 (1.13-2.47)	1.73 (1.18-2.60)	9

All models were adjusted for age and sex at Tromsø7.

DE, direct effect; ECG, electrocardiogram; HR, hazard ratio; IE, indirect effect; NA, not applicable; TE, total effect.

a HRs and 95% CIs were obtained from counterfactual natural-effects models.

b δ-age = predicted ECG age – chronological age.

c IE denotes the natural IE operating through δ-age; DE is the natural DE of each cluster independent of δ-age; TE is IE × DE. The proportion mediated (%) is calculated on the log-HR scale as ln(IE)/ln(TE) × 100.

d Negative, undefined or >100% values indicate inconsistent mediation.

### Association Between Trajectory Clusters and δ-Age

Figure 2A showed an association between the clusters and δ-age. Compared with the reference low-risk stable group, cluster 1, δ-age was on average 0.76 years (95% CI, 0.32-1.21) higher in cluster 2, 1.34 years (95% CI, 0.89-1.78) higher in cluster 3, and 1.61 years (95% CI, 1.09-2.13) higher in cluster 4, after adjustment for sex and baseline age.

**Figure 2:**
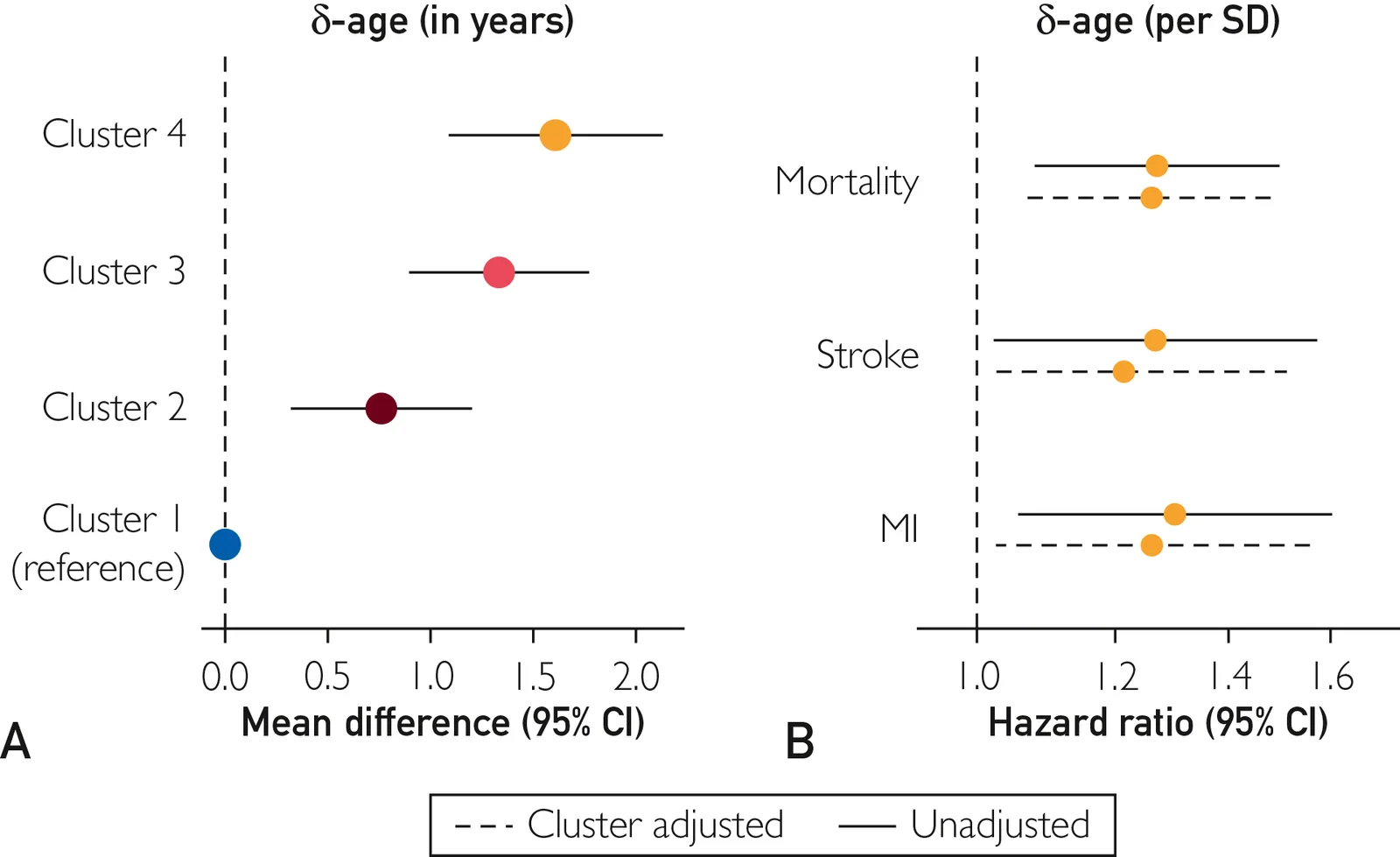
Association of accelerated heart aging (δ-age) with cardiovascular risk factor trajectories and clinical outcomes, The Tromsø Study 1986-2016, follow-up of clinical outcomes until 2021. (A) Mean difference in δ-age with 95% CIs across the 4 joint-trajectory clusters of body mass index, systolic blood pressure, diastolic blood pressure, and non–high-density lipoprotein (HDL) cholesterol. (B) Hazard ratios with 95% CIs for incident MI, incident stroke, and all-cause mortality per 1-SD increase in δ-age. All models were adjusted for sex and baseline chronological age.

### Association of δ-Age With Outcomes

Across the whole cohort (Figure 2B), each 1 SD increase in δ-age was associated with a 30% higher hazard of MI (HR, 1.30; 95% CI, 1.06-1.60), a 27% higher hazard of stroke (HR, 1.27; 95% CI, 1.02-1.58), and a 27% higher hazard of all-cause mortality (HR, 1.27; 95% CI, 1.08-1.50), independently of sex and chronological age. These HRs were slightly weakened after adjusting for the trajectory clusters.

### Association of Trajectory Cluster With Clinical End Points Mediated by δ-Age

Chronological age and sex-adjusted Cox proportional hazard models for the association between the clusters and end points are shown in Figure 3 and agree with TEs shown in Table 2. Relative to the low-risk reference, cluster 1, the total effect of the elevated non-HDL group (cluster 2) on MI was 1.22 (95% CI, 0.69-2.22), without reaching statistical significance. However, the hypertensive group (cluster 3) and high-risk group (cluster 4) showed 96% and 99% increased hazard of MI (HR, 1.96; 95% CI, 1.19-3.56 and HR, 1.99; 95% CI, 1.13-3.78, respectively). For stroke, clusters 3 and 4 showed two-fold increased total effects of incident stroke (HR, 2.04; 95% CI, 1.22-3.53 and HR, 2.40; 95% CI, 1.44-4.31, respectively). For all-cause mortality, only cluster 4 was statistically significant (HR, 1.73; 95% CI, 1.18-2.60) compared with the reference, cluster 1.

**Figure 3:**
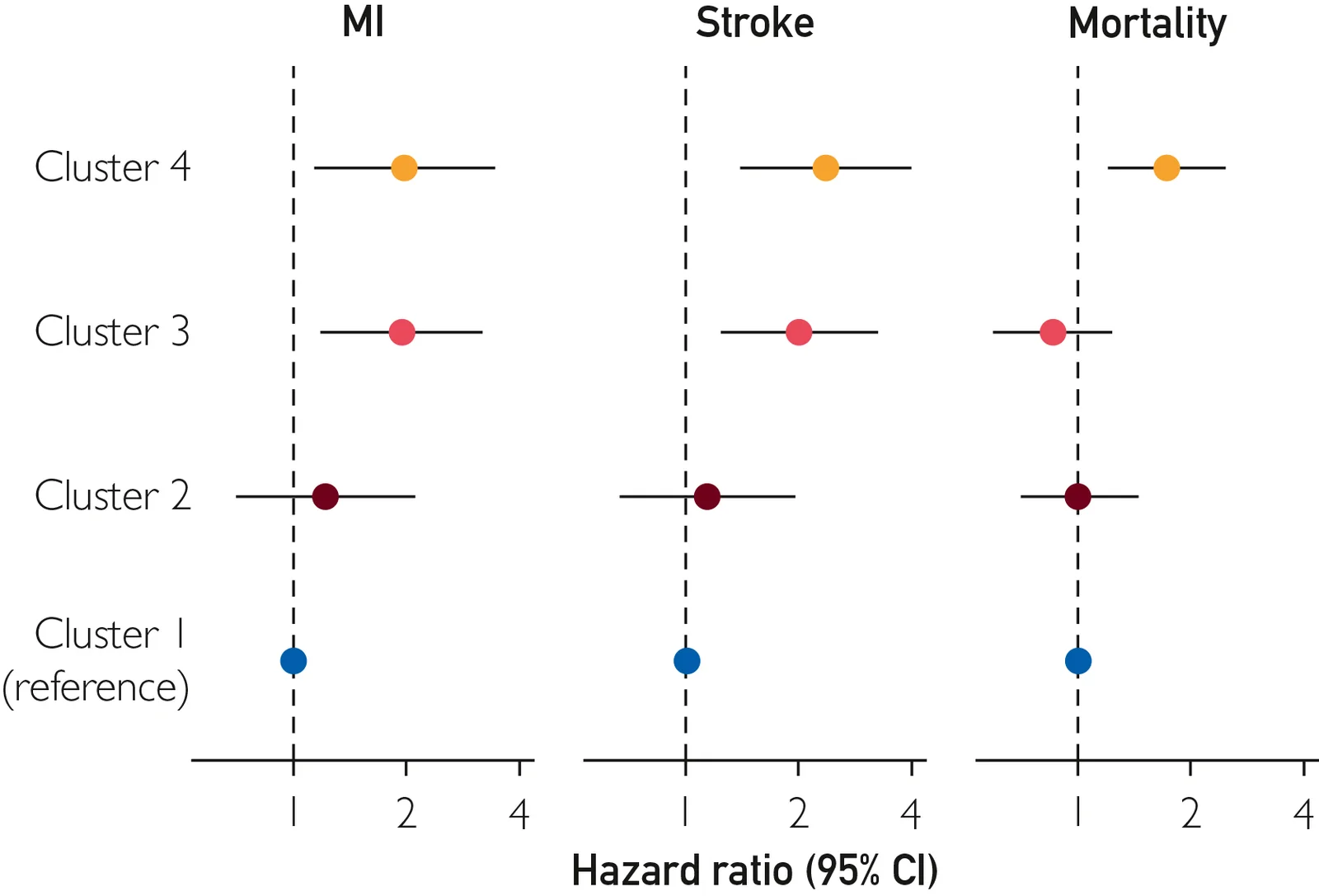
Hazard ratios and 95% CIs for MI, stroke, and all-cause mortality according to clusters 2 to 4 with cluster 1 as reference (stable low-risk profile). The Tromsø Study 1986-2016, follow-up of clinical outcomes until 2021.

Natural-effect models showed that the excess risk carried by the adverse trajectory clusters was explained largely by direct pathways, with biological aging (δ-age) accounting for only a small fraction of the associations (Table 2). For MI, the total effects in the hypertensive cluster 3 and high-risk trajectory cluster 4 were driven almost entirely by a DE (1.89; 95% CI, 1.13-3.42, and 1.89; 95% CI, 1.08-3.60, respectively). IEs were modest and not statistically significant (1.04; 95% CI, 0.97-1.12, and 1.05; 95% CI, 0.97-1.15, respectively), accounting for 6% and 7% of the TEs, respectively. A comparable picture was observed for stroke, where the DEs were statistically significant in clusters 3 and 4, with HRs of 1.97 (95% CI, 1.19-3.40) and 2.32 (95% CI, 1.42-4.17), respectively, and the corresponding IEs were near null (1.04; 95% CI, 0.96-1.12, and 1.04; 95% CI, 0.95-1.13, respectively), mediating only 6% and 4% of the TEs, respectively. For all-cause mortality, only the high-risk trajectory group, cluster 4, had a statistically significant DE (1.65; 95% CI, 1.13-2.47), with a modest and not statistically significant IE (1.05; 95% CI, 0.99-1.11), indicating no clear evidence that accelerated biological aging mediates mortality or cardiovascular event risk across clusters.

The supplementary analyses supported the main findings. Supplemental Figure 2 (available online at https://www.mcpdigitalhealth.org/) shows the age-standardized quadratic B-spline curves for systolic BP, diastolic BP, body mass index, and non-HDL cholesterol over 30 years of follow-up, illustrating the distinct patterns underlying clusters identified separately for each individual risk factor. Mediation analyses using these single-variable trajectory clusters, presented in Supplemental Table 1 (available online at https://www.mcpdigitalhealth.org/), yielded statistically nonsignificant IEs, in line with the main analysis. Supplemental Table 2 (available online at https://www.mcpdigitalhealth.org/) likewise showed limited mediation by δ-age when the associations were examined for risk factors measured at a single time point (Tromsø7). Repeating the clustering and mediation analyses with 3 (Supplemental Figure 3, https://www.mcpdigitalhealth.org/) and 5 cluster (Supplemental Figure 4, https://www.mcpdigitalhealth.org/) solutions produced comparable results, with small, statistically nonsignificant IEs through δ-age (Supplemental Tables 3 and 4, https://www.mcpdigitalhealth.org/).

## Discussion

Over 30 years of exposure, we identified 4 distinct trajectories of midlife cardiovascular risk factors (systolic and diastolic BP, BMI, and non-HDL cholesterol). These trajectory groups distinguished a stable low-risk profile from progressively adverse profiles and were strongly associated with incident MI, stroke, and all-cause mortality. The ECG-derived δ-age was an independent predictor of MI, stroke, or death, where each 1-SD increment in δ-age raised the risk by 27% to 30%. However, mediation analysis showed that δ-age mediated only a modest or statistically nonsignificant fraction of the excess risk carried by adverse risk factor trajectories. This implies that δ-age serves primarily as an independent indicator of accumulated cardiovascular wear, summarizing subclinical damage rather than acting as a causal intermediary between long-term risk exposures and clinical outcomes.

These results are consistent with prior work showing that δ-age reflects subclinical cardiovascular damage and is manifested later as diseases.^8^, ^10^, ^12^, ^14^ Our results also indicate that δ-age is only partly explained by the lifelong exposure to elevated BP, adiposity, and lipids, whereas a major component is likely explained by genetic, psychological, or environmental factors. Attia et al^8^ first showed that deep learning on 12-lead ECGs can predict a person’s biological age and suggested that a higher predicted age may flag poorer health. Subsequent large cohorts confirmed that positive δ-age independently forecasts cardiovascular risk.^10^, ^14^, ^27^ Consistent with previous research in the Tromsø Study, δ-age remained a significant predictor of MI and stroke even after adjustment for baseline risk factors.^28^, ^29^ We extended these observations by considering δ-age in the context of long-term risk factor trajectories. Longitudinal evidence repeatedly shows that chronically elevated BP, adiposity, and lipids from youth accelerate cardiovascular aging and risk. For instance, Wang et al.^30^ found that persistently high childhood trajectories of systolic BP and BMI predicted greater arterial stiffness and carotid intima-media thickness in midlife, while Niedermayer et al.^17^ identified distinct long-term cardiometabolic risk profiles associated with markedly different event rates. Consistent with this, participants in our worsening risk clusters had higher mean δ-age, suggesting that ECG-derived age captures the cumulative burden of decades of exposures.

Adjusting for δ-age only modestly attenuates the association between adverse trajectories and events, implying that the bulk of excess risk from sustained hypertension, dyslipidemia, or obesity operates through direct pathophysiological pathways (eg, prolonged hemodynamic stress, endothelial dysfunction, and atherogenesis) not fully captured by ECG-based age. In the broader biological aging literature, several studies have reported only partial mediation by aging biomarkers. For example, epigenetic age measures, particularly GrimAge, mediated approximately 30% to 40% of the association between Life’s Essential 8 cardiovascular health and incident CVD/mortality in certain subgroups,^31^ while a recent US cohort analysis found that PhenoAge and KDM-Age mediated approximately 20% to 22% of the Life’s Essential 8–mortality association.^32^ One plausible explanation for our modest δ-age mediation is that major risk factors act through multiorgan vascular injury, including the cerebral microvasculature (hypertension-related small-vessel disease and blood–brain barrier disruption^33^, ^34^) and carotid/large artery atherosclerosis (lipid-driven plaque initiation and progression^35^, ^36^), pathways that influence stroke and mortality but may be only partly reflected in an ECG-derived, heart aging metric. The near null IEs may simply reflect an absence of true mediation, with δ-age acting as a parallel marker of accumulated cardiovascular wear rather than a step on the causal pathway from risk factor trajectories to events. Furthermore, δ-age was measured once, at a mean age of approximately 66 years and decades after the exposure window opened, so it indexes concurrent cardiac state rather than the accumulating process itself. A single ECG carries appreciable within-person variability, and nondifferential measurement error in a mediator attenuates the IE toward the null while leaving the DE comparatively intact. Categorical cluster membership also discards within-cluster variation in cumulative burden. Finally, natural-effects models assume no exposure-induced mediator-outcome confounding, an assumption that interim medication use may violate. At the same time, it is notable that this single, inexpensive ECG-based snapshot obtained later in life appears to capture part of the risk information carried by the long-term risk factors, which are seldom available in routine clinical settings.

In summary, decades-long risk factor trajectories refine cardiovascular risk stratification, whereas δ-age adds complementary, patient-specific prognostic insight as a biomarker of cardiovascular aging. Yet, the mechanistic drivers of MI, stroke, and mortality remain largely the well-known culprits of sustained hypertension, dyslipidemia, and obesity over time. For clinicians, δ-age is very informative, as it is inexpensive and noninvasive and summarizes accumulated cardiovascular aging from a single snapshot ECG, independently of age, sex, and traditional risk factor levels. Because it mediates little of the risk factors, an elevated value should prompt attention even when the BP, adiposity, and lipids are in the reference range. Nevertheless, modifying the underlying risk factor trajectories remains paramount for reducing cardiovascular events.^8^, ^10^, ^30^

### Strengths and Limitations

The Tromsø Study’s 5 repeated examination waves (1986-2016) provide a longitudinal, prospective framework, allowing us to disentangle temporal patterns from between-person differences. Participation has remained high at each wave, minimizing selection and information bias. Rigorous, centrally standardized protocols for BP measurement, venepuncture, and biochemical analyses further reduce measurement error. Age dependent trends were removed through fractional polynomial standardization, and the cohort is linked to virtually complete, adjudicated national registries for MI, stroke, and all-cause mortality, ensuring precise outcome ascertainment and eliminating loss to follow up.

One of the limitations of this study is that the δ-age metric was derived from the CNN trained on Mayo hospital patients ECG data. It may reflect pathological rather than healthy aging, therefore may not be fully applicable to a community cohort. Since δ-age was available only in Tromsø7, it was not possible to model a withinperson change and any consequences for the mediation estimates. Our clustering assumes roughly spherical, equal variance clusters and can be sensitive to the chosen number of clusters. Consequent misclassification of individuals may bias cluster outcome associations and mediation estimates. In addition, the relatively small number of clinical events may have limited statistical power. Our cohort is almost exclusively of Northern European population and drawn from a single municipality, which may limit generalizability to more diverse populations. Finally, although our statistical approach adjusts for confounders, residual confounding (eg, by diet or psychosocial factors not repeatedly measured) cannot be completely excluded.

## Conclusion

Accelerated cardiovascular aging (δ-age) emerges as a potent individual level biomarker complementing traditional risk factors. Its strong direct associations with a 27% to 30% higher risk of MI, stroke, and mortality per 1-SD increment in δ-age, together with its minimal 4% to 9% mediation of risk factor trajectory effects through δ-age, indicate that δ-age captures additional pathophysiology not reflected by classic risk factors. Because δ-age can be measured noninvasively from a standard ECG, future studies should test whether it could serve as a scalable, personalized tool for risk stratification that identifies high-risk individuals overlooked by standard approaches. Further work should also establish whether serial ECGs can capture within person change in δ-age, whether δ-age responds to modification of the underlying risk factors, and whether these findings replicate in ethnically diverse cohorts and in populations with a higher burden of clinical events.

## Potential Competing Interests

Dr Lopez-Jimenez reports serving as principal investigator in a multicenter trial testing implementation of an AI ECG algorithm to detect low ejection fraction, sponsored by Anumana, and receiving research support from DMI. Mayo Clinic has licensed the rights to commercialize the algorithm to Anumana, and Dr Lopez-Jimenez is a coinventor of the algorithm used in the manuscript that has been licensed to Anumana. Mayo Clinic and Dr Lopez-Jimenez may benefit financially in the future if commercialization occurs; however, he has not received royalties or other financial compensation related to this algorithm to date. Dr Lopez-Jimenez also reports serving on scientific advisory boards for Anumana, Novo Nordisk, Ionis, New Amsterdam, K-Health, Kento Health, Wizehealth, and Ultrasight; serving as director of an educational program for Novo Nordisk employees, with royalties paid to Mayo Clinic; being a coinventor on a patent related to an AI ECG algorithm to detect low biological age by ECG; and serving as Chair Elect of the Obesity Council of the American Heart Association, uncompensated. Dr Schirmer reports consulting fees from Pharma Cosmos, Bayer, and Novartis; payment or honoraria for lectures, presentations, or educational events from AstraZeneca, Biogen, Boehringer, Sanofi, and Novo Nordisk; has a pending patent for an algorithm for enhancing detection of valvular heart disease by auscultation; and unpaid roles as a member of the working group on preventive cardiology of the Norwegian Society of Cardiology, a member of the Norwegian Health Association Research Council for Dementia (Chair 2023-2024), and a member of the editorial board Norwegian Pharmacopeia. Given their roles as Editor and Editorial Board Member, Dr Lopez-Jimenez and Dr Attia had no involvement in the peer-review of this article and had no access to information regarding its peer-review. Full responsibility for the editorial process for this article was delegated to an unaffiliated editor. All other authors report no competing interests.

## Ethics Statement

The Tromsø Study complies with the Declaration of Helsinki and has been approved by the Regional Committee for Medical and Health Research Ethics (REK), the Data Inspectorate, and the Norwegian Directorate of Health. All participants provided written informed consent. The current study was approved by the REK Nord (reference 68185), and a data management plan was evaluated by the Norwegian Centre for Research Data.

## Supplemental Online Material

STROBE-checklist-v4
