# Supplementary material for: Clustering of Cardiovascular Risk Factors in Midlife Forms Trajectories That Affect Heart Aging Biomarker and Incidence of Cardiovascular Disease
Source: Mayo Clin Proc Digit Health. 2026 Oct 5;4(4):100425. doi: 10.1016/j.mcpdig.2026.100425 (PMC13718786; doi:10.1016/j.mcpdig.2026.100425)
Supplement: Supplementary file 1 [file mmc2.docx]

**Supplementary Methods**

**Measurement of cardiovascular risk factors**

Systolic and diastolic blood pressure was measured on the participant’s right upper arm with a properly sized cuff based on arm circumference, all measurements being performed by trained personnel. Blood pressure was measured three times with an oscillometric digital automatic device (in Tromsø3-5: Dinamap Vital Signs Monitor; Critikon Inc, Tampa, FL, and in Tromsø6 and 7: Dinamap ProCare 300 monitor, GE Healthcare, Oslo, Norway), measurements being separated by a one-minute interval after a two-minute seated rest.

Non-fasting venous blood samples were collected by standard methods by trained personnel, with the participant sitting. Serum total cholesterol, high density lipoprotein (HDL) cholesterol and triglycerides were analysed by enzymatic colorimetric methods with Hitachi 737 (Boehringer-Mannheim, Mannheim, Germany) (Tromsø3 and 4), Hitachi 917 (Tromsø5), Modular PPE (Tromsø6) and Cobas 8000 c702 (Tromsø7) (all: Roche Diagnostics, Mannheim, Germany). HDL cholesterol was measured after precipitation of lower density lipoprotein with heparin and manganese chloride (Tromsø3 and 4) or directly (Tromsø5-7).

**Ascertainment of clinical end-points**

The local cardiovascular disease register was formed by linkage to diagnoses registered at the University Hospital of North Norway and the National Causes of Death Registry. All Norwegian hospitals are mandated to register patients hospitalized with acute myocardial infarctions to the Norwegian Myocardial Infarction Register and acute strokes to the Norwegian Stroke Register since 2012. Myocardial infarction inclusion criteria were patients with an I21 or I22 diagnosis hospitalized within 28 days after symptom onset; for stroke, those admitted with I61, I63, or I64. The Stroke Register did not include strokes following a traumatic head injury, stroke related to intracranial tumors, or ischemic stroke following a subarachnoid haemorrhage.

**Age standardisation of risk-factor variables**

For each variable and study wave, the best one- or two-term fractional polynomial model of chronological age was chosen by the minimum Akaike information criterion. The fitted mean and variance functions provided smoothed, age-dependent reference values, and residuals were divided by the model-based age-specific SD to obtain standardised scores.

**Trajectory derivation and mediation modelling**

The K-means algorithm converged when less than 0.01% of participants changed cluster membership or when the relative reduction in within-cluster error was less than 10⁻⁷. In the natural-effects models, the total effect was obtained by summing the log-HRs of the direct and indirect components and then exponentiating this sum to yield the overall hazard ratio.

Supplemental Figure 1 Cumulative incidence of myocardial infarction, stroke, and all-cause mortality according to multivariable cardiovascular risk-factor trajectory clusters:
Cumulative incidence curves are shown for **(A)** incident myocardial infarction, **(B)** incident stroke, and **(C)** all-cause mortality during follow-up from Tromsø7 baseline, 2015–2016, through 2021. The four trajectory clusters were derived from 30-year joint longitudinal patterns of body mass index, systolic blood pressure, diastolic blood pressure, and non-HDL cholesterol using age-standardised quadratic B-spline trajectories and K-means clustering. Cluster 1 represents the stable low-risk group, Cluster 2 the dyslipidemia group, Cluster 3 the hypertension group, and Cluster 4 the high-risk group. Solid lines show cumulative incidence estimates, and shaded areas indicate 95% confidence intervals. The x-axis represents years since baseline at Tromsø7.

**Supplementary analyses of single-variable longitudinal trajectory clusters, associations with clinical outcomes, and mediation by δ-age**

Supplemental Figure 2: Age-standardised quadratic B-spline trajectories of (A) systolic blood pressure (SBP), (B) diastolic blood pressure (DBP), (C) body mass index (BMI), and (D) non-HDL cholesterol over 30 years of follow-up, with clusters derived separately for each individual risk factor. Panels show the fitted spline curves for cluster 1, cluster 2, cluster 3, and cluster 4 for each risk factor.

Supplemental Table 1: Natural-effect decomposition^a^ of the associations between univariate trajectory clusters (K = 4) of SBP, DBP, BMI and non-HDL and incident myocardial infarction, stroke, and all-cause mortality, with δ-age^b^ (predicted ECG age – chronological age) specified as the mediator; The Tromsø Study 2015–2016, follow-up through 2021.

| Exposure | Cluster number, N (%) | | Events N (%) | IE^c^ (95 % CI) | DE^c^ (95 % CI) | TE^c^ (95 % CI) | Proportion Mediated (%) |
| --- | --- | --- | --- | --- | --- | --- | --- |
| Myocardial infarction | |  |  |  |  |  |  |
| SBP | 1, 1659 (31.6) | | 31 (1.9%) | 1 (Reference) | 1 (Reference) | 1 (Reference) | (Reference) |
|  | 2, 1354 (25.8) | | 25 (1.8%) | 1.042 (0.971, 1.121) | 0.896 (0.505, 1.511) | 0.934 (0.524, 1.576) | NA^d^ |
|  | 3, 1318 (25.1) | | 32 (2.4%) | 1.023 (0.962, 1.093) | 0.932 (0.560, 1.543) | 0.953 (0.576, 1.566) | NA |
|  | 4, 913 (17.4) | | 49 (5.4%) | 1.049 (0.975, 1.133) | 2.220 (1.417, 3.570) | 2.328 (1.506, 3.809) | 5.6 |
| DBP | 1, 1483 (28.3) | | 29 (2.0%) | 1 (Reference) | 1 (Reference) | 1 (Reference) |  |
|  | 2, 1363 (26.0) | | 28 (2.1%) | 1.042 (0.969, 1.133) | 0.760 (0.436, 1.319) | 0.792 (0.454, 1.363) | NA |
|  | 3, 1351 (25.8) | | 30 (2.2%) | 1.044 (0.963, 1.132) | 0.905 (0.541, 1.531) | 0.945 (0.554, 1.620) | NA |
|  | 4, 1047 (20.0) | | 50 (4.8%) | 1.076 (0.989, 1.183) | 1.573 (0.967, 2.639) | 1.693 (1.037, 2.824) | 14 |
| BMI | 1, 1567 (29.9) | | 42 (2.7%) | 1 (Reference) | 1 (Reference) | 1 (Reference) | (Reference) |
|  | 2, 2117 (40.4) | | 51 (2.4%) | 1.010 (0.950, 1.071) | 0.674 (0.432, 1.041) | 0.681 (0.436, 1.047) | NA |
|  | 3, 1205 (23.0) | | 29 (2.4%) | 1.028 (0.960, 1.103) | 0.631 (0.392, 1.020) | 0.649 (0.407, 1.027) | NA |
|  | 4, 355 (6.8) | | 15 (4.2%) | 1.048 (0.951, 1.159) | 1.265 (0.666, 2.279) | 1.325 (0.693, 2.400) | 16.7 |
| non-HDL | 1, 1543 (29.4) | | 23 (1.5%) | 1 (Reference) | 1 (Reference) | 1 (Reference) | (Reference) |
|  | 2, 1864 (35.5) | | 49 (2.6%) | 1.002 (0.944, 1.066) | 1.837 (1.097, 3.188) | 1.841 (1.134, 3.182) | 0.3 |
|  | 3, 918 (17.5) | | 35 (3.8%) | 1.038 (0.960, 1.126) | 2.030 (1.189, 3.726) | 2.107 (1.226, 3.892) | 5.0 |
|  | 4, 919 (17.5) | | 30 (3.3%) | 0.996 (0.923, 1.068) | 1.989 (1.149, 3.701) | 1.981 (1.171, 3.651) | NA |
| Stroke | | |  |  |  |  |  |
| SBP | 1, 1659 (31.6) | | 27 (1.6%) | 1 (Reference) | 1 (Reference) | 1 (Reference) | (Reference) |
|  | 2, 1354 (25.8) | | 44 (3.2%) | 1.030 (0.959, 1.104) | 1.849 (1.145, 3.099) | 1.904 (1.166, 3.165) | 4.6 |
|  | 3, 1318 (25.1) | | 31 (2.4%) | 1.023 (0.956, 1.101) | 1.336 (0.761, 2.365) | 1.367 (0.792, 2.410) | 7.3 |
|  | 4, 913 (17.4) | | 32 (3.5%) | 1.048 (0.957, 1.145) | 2.042 (1.205, 3.354) | 2.140 (1.277, 3.583) | 6.2 |
| DBP | 1, 1483 (28.3) | | 20 (1.3%) | 1 (Reference) | 1 (Reference) | 1 (Reference) | (Reference) |
|  | 2, 1363 (26.0) | | 46 (3.4%) | 1.028 (0.954, 1.100) | 2.535 (1.525, 4.538) | 2.605 (1.539, 4.809) | 2.9 |
|  | 3, 1351 (25.8) | | 29 (2.1%) | 1.036 (0.960, 1.123) | 1.577 (0.889, 2.895) | 1.633 (0.891, 3.048) | 7.2 |
|  | 4, 1047 (20.0) | | 39 (3.7%) | 1.062 (0.959, 1.174) | 2.815 (1.665, 5.025) | 2.990 (1.773, 5.361) | 5.5 |
| BMI | 1, 1567 (29.9) | | 38 (2.4%) | 1 (Reference) | 1 (Reference) | 1 (Reference) | (Reference) |
|  | 2, 2117 (40.4) | | 49 (2.3%) | 1.005 (0.950, 1.069) | 0.934 (0.612, 1.500) | 0.939 (0.610, 1.541) | NA |
|  | 3, 1205 (23.0) | | 37 (3.1%) | 1.015 (0.950, 1.083) | 1.204 (0.754, 1.936) | 1.222 (0.765, 2.001) | 7.4 |
|  | 4, 355 (6.8) | | 10 (2.8%) | 1.023 (0.922, 1.135) | 1.092 (0.465, 2.180) | 1.117 (0.476, 2.265) | 20.7 |
| non-HDL | 1, 1543 (29.4) | | 32 (2.1%) | 1 (Reference) | 1 (Reference) | 1 (Reference) | (Reference) |
|  | 2, 1864 (35.5) | | 55 (3.0%) | 0.997 (0.937, 1.061) | 1.382 (0.912, 2.179) | 1.378 (0.901, 2.172) | NA |
|  | 3, 918 (17.5) | | 18 (2.0%) | 1.023 (0.947, 1.111) | 0.824 (0.438, 1.498) | 0.843 (0.450, 1.547) | NA |
|  | 4, 919 (17.5) | | 29 (3.2%) | 0.994 (0.924, 1.068) | 1.426 (0.856, 2.336) | 1.418 (0.854, 2.357) | NA |
| All-cause mortality | | |  |  |  |  |  |
| SBP | 1, 1659 (31.6) | | 71 (4.3%) | 1 (Reference) | 1 (Reference) | 1 (Reference) | (Reference) |
|  | 2, 1354 (25.8) | | 47 (3.5%) | 1.037 (0.984, 1.098) | 0.726 (0.485, 1.054) | 0.752 (0.508, 1.093) | NA |
|  | 3, 1318 (25.1) | | 72 (5.5%) | 1.029 (0.979, 1.083) | 1.097 (0.785, 1.524) | 1.128 (0.809, 1.573) | 23.4 |
|  | 4, 913 (17.4) | | 42 (4.6%) | 1.056 (0.986, 1.128) | 0.941 (0.640, 1.369) | 0.994 (0.673, 1.430) | NA |
| DBP | 1, 1483 (28.3) | | 59 (4.0%) | 1 (Reference) | 1 (Reference) | 1 (Reference) | (Reference) |
|  | 2, 1363 (26.0) | | 48 (3.5%) | 1.038 (0.978, 1.100) | 0.755 (0.509, 1.116) | 0.784 (0.519, 1.159) | NA |
|  | 3, 1351 (25.8) | | 77 (5.7%) | 1.049 (0.990, 1.117) | 1.284 (0.896, 1.801) | 1.347 (0.933, 1.871) | 16.0 |
|  | 4, 1047 (20.0) | | 48 (4.6%) | 1.080 (1.000, 1.167) | 0.918 (0.617, 1.369) | 0.992 (0.665, 1.472) | NA |
| BMI | 1, 1567 (29.9) | | 60 (3.8%) | 1 (Reference) | 1 (Reference) | 1 (Reference) |  |
|  | 2, 2117 (40.4) | | 90 (4.3%) | 1.005 (0.961, 1.053) | 1.025 (0.737, 1.447) | 1.030 (0.744, 1.455) | 16 |
|  | 3, 1205 (23.0) | | 53 (4.4%) | 1.016 (0.967, 1.068) | 1.007 (0.684, 1.472) | 1.023 (0.696, 1.517) | 69.1 |
|  | 4, 355 (6.8) | | 29 (8.2%) | 1.036 (0.960, 1.112) | 2.002 (1.230, 3.082) | 2.074 (1.251, 3.234) | 4.8 |
| non-HDL | 1, 1543 (29.4) | | 65 (4.2%) | 1 (Reference) | 1 (Reference) | 1 (Reference) | (Reference) |
|  | 2, 1864 (35.5) | | 61 (3.3%) | 0.997 (0.950, 1.042) | 0.743 (0.523, 1.069) | 0.741 (0.521, 1.066) | 1.1 |
|  | 3, 918 (17.5) | | 62 (6.8%) | 1.023 (0.966, 1.083) | 1.360 (0.939, 1.977) | 1.392 (0.954, 2.017) | 7.0 |
|  | 4, 919 (17.5) | | 44 (4.8%) | 0.992 (0.931, 1.051) | 1.016 (0.672, 1.503) | 1.008 (0.667, 1.490) | NA |

All models were adjusted for age and sex at Tromsø7.
a Hazard ratios (HRs) and 95 % confidence intervals (CI) were obtained from counterfactual natural-effects models.
b δ-age = predicted ECG age – chronological age
c IE denotes the natural indirect effect operating through δ-age; DE is the natural direct effect of each cluster independent of δ-age; TE is the total effect (IE × DE). The proportion mediated (%) is calculated on the log-HR scale as ln(IE)/ln(TE) × 100.
d NA, not applicable: Negative or >100 % values indicate inconsistent mediation.

**Supplementary analysis of risk factors measured at a single time point (Tromsø7), their associations with clinical outcomes, and mediation by δ-age**

Supplemental Table 2: Natural‐effect decomposition^a^ of the association between traditional risk factor measured at a single timepoint (Tromsø7) and incident cardiovascular outcomes, with δ-age^b^ specified as the mediator. The Tromsø Study 2015-2016, follow-up of clinical outcomes until 2021.

|  | IE^c^ (95 % CI) | DE^c^ (95 % CI) | TE^c^ (95 % CI) | Proportion Mediated (%) |
| --- | --- | --- | --- | --- |
| Myocardial infarction | | | | |
| SBP | 1.001 (0.999, 1.003) | 1.008 (0.998, 1.018) | 1.009 (0.999, 1.019) | 11.16 |
| DBP | 1.001 (0.997, 1.006) | 1.010 (0.990, 1.031) | 1.011 (0.991, 1.032) | 9.14 |
| BMI | 1.003 (0.994, 1.014) | 0.992 (0.942, 1.043) | 0.995 (0.944, 1.047) | NA^d^ |
| Non-HDL | 0.996 (0.961, 1.033) | 1.190 (1.013, 1.395) | 1.185 (1.016, 1.382) | NA |
| Stroke | | | | |
| SBP | 1.001 (0.999, 1.003) | 1.011 (1.002, 1.019) | 1.012 (1.003, 1.020) | 8.38 |
| DBP | 1.001 (0.997, 1.005) | 1.042 (1.023, 1.061) | 1.043 (1.025, 1.063) | 2.37 |
| BMI | 1.002 (0.993, 1.011) | 0.998 (0.953, 1.043) | 0.999 (0.956, 1.043) | NA |
| Non-HDL | 0.999 (0.965, 1.039) | 1.279 (1.085, 1.504) | 1.279 (1.078, 1.495) | NA |
| All-cause mortality | | | | |
| SBP | 1.001 (0.999, 1.002) | 0.992 (0.985, 1.000) | 0.993 (0.985, 1.000) | NA |
| DBP | 1.001 (0.998, 1.005) | 0.981 (0.967, 0.995) | 0.983 (0.969, 0.997) | NA |
| BMI | 1.002 (0.995, 1.010) | 1.010 (0.971, 1.048) | 1.012 (0.974, 1.049) | 16.75 |
| Non-HDL | 0.997 (0.969, 1.028) | 0.936 (0.820, 1.071) | 0.934 (0.819, 1.062) | 4.4 |

All models were adjusted for age and sex at Tromsø7.
a. Hazard ratios (HRs) and 95 % confidence intervals (CI) were obtained from counterfactual natural-effects models.
b. δ-age = predicted ECG age – chronological age
c. IE denotes the natural indirect effect operating through δ-age; DE is the natural direct effect of each cluster independent of δ-age; TE is the total effect (IE × DE). The proportion mediated (%) is calculated on the log-HR scale as ln(IE)/ln(TE) × 100.
d. NA, not applicable: Negative or >100 % values indicate inconsistent mediation.

**Sensitivity analyses of the joint multivariable trajectory clustering using three- and five-cluster solutions, associations with clinical outcomes, and mediation by δ-age**

Supplemental Figure 3: Age-standardised quadratic B-spline trajectories of (A) systolic blood pressure (SBP), (B) diastolic blood pressure (DBP), (C) body mass index (BMI), and (D) non-HDL cholesterol over 30 years of follow-up, for the three-cluster solution of the joint multivariable trajectory clustering. Panels show the fitted spline curves for cluster 1, cluster 2 and cluster 3 for each risk factor. The Tromsø Study 1986–2016.

Supplemental Table 3: Natural-effect decomposition^a^ of the associations between the three-cluster solution of the joint multivariable trajectory clusters and incident myocardial infarction, stroke, and all-cause mortality, with δ-age^b^ specified as the mediator. The Tromsø Study 2015–2016, follow-up through 2021.

| Cluster number, N (%) | Events N (%) | IE^c^ (95 % CI) | DE^c^ (95 % CI) | TE^c^ (95 % CI) | Proportion Mediated (%) |  |
| --- | --- | --- | --- | --- | --- | --- |
|  |  |  |  |  |  |  |
| Myocardial infarction | | | | | | |
| 1, 1269 (24.2) | 38 (3.0) | 1 (Reference) | 1 (Reference) | 1 (Reference) | (Reference) |  |
| 2, 2265 (43.2) | 36 (1.6) | 0.96 (0.89, 1.03) | 0.68 (0.42, 1.05) | 0.65 (0.41, 1.00) | 9 |  |
| 3, 1710 (32.6) | 63 (3.7) | 1.00 (0.93, 1.06) | 1.19 (0.8, 1.78) | 1.19 (0.79, 1.80) | 0 |  |
| Stroke | | | | | | |
| 1, 1269 (24.2) | 40 (3.2) | 1 (Reference) | 1 (Reference) | 1 (Reference) | (Reference) |  |
| 2, 2265 (43.2) | 39 (1.7) | 0.97 (0.90, 1.04) | 0.57 (0.36, 0.91) | 0.55 (0.36, 0.88) | 5 |  |
| 3, 1710 (32.6) | 55 (3.2) | 1.01 (0.94, 1.07) | 1.01 (0.65, 1.58) | 1.01 (0.66, 1.59) | NA^d^ |  |
| All-cause mortality | | | | | | |
| 1, 1269 (24.2) | 76 (6.0) | 1 (Reference) | 1 (Reference) | 1 (Reference) | (Reference) |  |
| 2, 2265 (43.2) | 88 (3.9) | 0.96 (0.91, 1.01) | 0.73 (0.54, 1.00) | 0.70 (0.52, 0.96) | 11 |  |
| 3, 1710 (32.6) | 68 (4.0) | 1.00 (0.95, 1.05) | 0.63 (0.46, 0.89) | 0.63 (0.46, 0.89) | 0 |  |

All models were adjusted for age and sex at Tromsø7.
a. Hazard ratios (HRs) and 95 % confidence intervals (CI) were obtained from counterfactual natural-effects models.
b. δ-age = predicted ECG age – chronological age
c. IE denotes the natural indirect effect operating through δ-age; DE is the natural direct effect of each cluster independent of δ-age; TE is the total effect (IE × DE). The proportion mediated (%) is calculated on the log-HR scale as ln(IE)/ln(TE) × 100.
d. NA, not applicable: Negative or >100 % values indicate inconsistent mediation

Supplemental Figure 4: Age-standardised quadratic B-spline trajectories of (A) systolic blood pressure (SBP), (B) diastolic blood pressure (DBP), (C) body mass index (BMI), and (D) non-HDL cholesterol over 30 years of follow-up, for the five-cluster solution of the joint multivariable trajectory clustering. Panels show the fitted spline curves for clusters 1 to 5 for each risk factor. The Tromsø Study 1986–2016.

Supplemental Table 4: Natural-effect decomposition^a^ of the associations between the five-cluster solution of the joint multivariable trajectory clusters and incident myocardial infarction, stroke, and all-cause mortality, with δ-age^b^ specified as the mediator. The Tromsø Study 2015–2016, follow-up through 2021.

| Cluster number, N (%) | Events N (%) | IE^c^ (95 % CI) | DE^c^ (95 % CI) | TE^c^ (95 % CI) | Proportion Mediated (%) |  |
| --- | --- | --- | --- | --- | --- | --- |
|  |  |  |  |  |  |  |
| Myocardial infarction | | | | | | |
| 1, 1221 (23.3) | 23 (1.9) | 1 (Reference) | 1 (Reference) | 1 (Reference) | (Reference) |  |
| 2, 1325 (25.3) | 38 (2.9) | 1.03 (0.96, 1.1) | 1.47 (0.89, 2.52) | 1.51 (0.91, 2.56) | 7 |  |
| 3, 642 (12.2) | 22 (3.4) | 1.04 (0.96, 1.14) | 1.74 (0.98, 2.98) | 1.81 (1.01, 3.13) | 7 |  |
| 4, 1233 (23.5) | 15 (1.2) | 0.98 (0.91, 1.06) | 0.88 (0.42, 1.75) | 0.86 (0.42, 1.71) | NA^d^ |  |
| 5, 823 (15.7) | 39 (4.7) | 1.04 (0.96, 1.12) | 2.14 (1.22, 3.83) | 2.22 (1.27, 3.95) | 5 |  |
| Stroke | | | | | | |
| 1, 1221 (23.3) | 28 (2.3) | 1 (Reference) | 1 (Reference) | 1 (Reference) |  |  |
| 2, 1325 (25.3) | 44 (3.3) | 1.03 (0.96, 1.1) | 1.43 (0.86, 2.37) | 1.46 (0.88, 2.46) | 8 |  |
| 3, 642 (12.2) | 18 (2.8) | 1.03 (0.95, 1.12) | 1.17 (0.62, 2.1) | 1.2 (0.63, 2.24) | 16 |  |
| 4, 1233 (23.5) | 18 (1.5) | 0.98 (0.92, 1.06) | 0.72 (0.37, 1.3) | 0.71 (0.36, 1.28) | NA |  |
| 5, 823 (15.7) | 26 (3.2) | 1.03 (0.95, 1.13) | 1.42 (0.79, 2.57) | 1.47 (0.82, 2.64) | 8 |  |
| All-cause mortality | | | | | | |
| 1, (1221 (23.3) | 53 (4.3) | 1 (Reference) | 1 (Reference) | 1 (Reference) |  |  |
| 2, 1325 (25.3) | 62 (4.7) | 1.03 (0.97, 1.09) | 1.04 (0.73, 1.53) | 1.07 (0.74, 1.59) | 44 |  |
| 3, 642 (12.2) | 44 (6.9) | 1.04 (0.97, 1.11) | 1.53 (1.01, 2.36) | 1.59 (1.05, 2.42) | 8 |  |
| 4, 1233 (23.5) | 42 (3.4) | 0.98 (0.93, 1.04) | 0.98 (0.65, 1.49) | 0.97 (0.64, 1.48) | NA |  |
| 5, 823 (15.7) | 31 (3.8) | 1.04 (0.98, 1.11) | 0.82 (0.51, 1.26) | 0.86 (0.53, 1.32) | NA |  |

All models were adjusted for age and sex at Tromsø7.
a. Hazard ratios (HRs) and 95 % confidence intervals (CI) were obtained from counterfactual natural-effects models.
b. δ-age = predicted ECG age – chronological age
c. IE denotes the natural indirect effect operating through δ-age; DE is the natural direct effect of each cluster independent of δ-age; TE is the total effect (IE × DE). The proportion mediated (%) is calculated on the log-HR scale as ln(IE)/ln(TE) × 100.
d. NA, not applicable: Negative or >100 % values indicate inconsistent mediation
